# Socket Shield Technique of an Ailing Mandibular Molar With Customized Healing Abutment: Graftless Management of the Hard and Soft Tissue Foundation Around Immediate Dental Implants

**DOI:** 10.1155/crid/9969134

**Published:** 2025-02-18

**Authors:** Sanjay Kumar Sah

**Affiliations:** Department of Prosthodontics, Bir Hospital, National Academy of Medical Sciences, Kathmandu, Nepal

**Keywords:** customized healing abutment, graftless implant, molar immediate implants, socket shield technique

## Abstract

It is an established fact that postextraction ridge resorptive changes are inevitable and are very evident in the molar areas. Resorption in the molar sites can cause a reduction in the attached gingiva and affect the long-term success of the osseointegrated implant. To prevent significant postextraction tissue alteration, the socket shield technique (SST) was developed to preserve the buccal plate, over a decade ago. Since then, various studies showcasing modifications of the technique have been published mainly focusing on SST in conjunction with immediate implants in the anterior esthetic zone. Gluckman gave a collective term called partial extraction therapy (PET) which includes SST, pontic shield technique, and root submergence technique. He suggested using a graft material in the gap between the shield and the implant. Later, Siormpas et al. advocated a root membrane technique (RMT) and suggested that it may not be necessary to use the graft material. With the evolution of the technique, the terms SST and RMT are more similar to each other now, with the only difference in the sequence of shield preparation and implant placement. The shield is prepared first, and osteotomy is done in the former and osteotomy is done before shield preparation in the latter. The SST technique is often ignored as a possibility in the molar sites. Though technique-sensitive, SST with immediate implants in molars with a customized healing abutment ensures the maintenance of the original hard and soft tissue volumes in the most conservative way. The following case report showcases a stepwise, graftless management approach for a nonrestorable right mandibular molar with SST and immediate implant. Long-term randomized controlled trials (RCTs) on molar SST are encouraged to make a recommendation for routine clinical practice.

## 1. Introduction

Dental implants have emerged as the gold standard for the replacement of missing or nonrestorable teeth, considered for extraction. The weighted mean changes during the postextraction healing period, based on the data derived from some selected studies, show the clinical loss in width to be greater than the loss in height, assessed both clinically as well as radiographically [1]. Human re-entry studies showed horizontal bone loss of 29%–63% and vertical bone loss of 11%–22% after 6 months following tooth extraction [2]. Several methods, such as immediate implants and guided bone regeneration (GBR), have been researched clinically to minimize this volumetric hard and soft tissue changes post extraction. A study by Wang and Lang concluded that implants placed into the fresh extraction sockets neither prevent the resorption of the alveolar bone, nor soft tissue grafts or primary closure did not show beneficial effect on preserving the alveolar bone [3]. To maintain soft and hard tissue contour and achieve long-term esthetic success, Hürzeler et al. published a proof-of-concept study of the socket shield technique (SST) which concluded that retaining the buccal aspect of the root during implant placement may be beneficial in preserving the buccal bone plate [4]. A meta-analysis confirmed that SST can reduce changes in buccal plate width and height and improve the soft tissue profile following immediate implant placement in the esthetic zone [5].

Whereas there is evidence in the literature that socket shield may prevent the resorption of labial plate [6, 7], there is a paucity of the data regarding healing of socket with socket shield with or without grafting. Pohl et al. [8] reported clinical observations in 34 sockets with a socket shield, either nongrafted or grafted with autologous materials. They found that soft tissue growth along the lingual surface of the socket shield was typical, except in cases where the sockets were grafted with nondemineralized autologous tooth dentin or cortical tuberosity bone plate.

Another study concluded that molar sites have larger postextraction dimensional changes, particularly in the horizontal dimension [9]. Thus, preserving the buccal plate for immediate implants in posterior sites provides a significant advantage for long-term success. The majority of the literature on SST with immediate implants is on the anterior maxilla focusing on the esthetic score. However, the following case report highlights graftless, hard and soft tissue management of a nonrestorable mandibular molar with SST and a customized healing abutment.

## 2. Case Report

**Figure 1:**
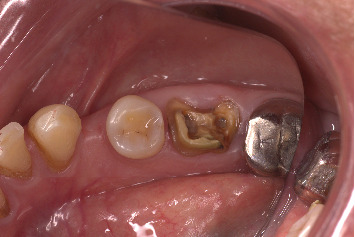
Nonrestorable right mandibular first molar.

**Figure 2:**
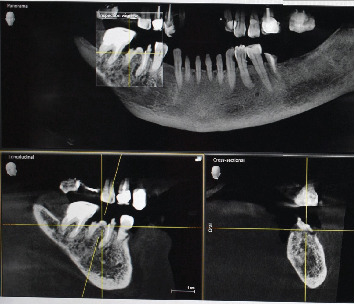
CBCT for treatment planning.

A 71-year-old systemically healthy patient reported with a nonrestorable right first mandibular molar (Figure 1). Radiographic investigation was done with a cone-beam computed tomography (CBCT) (Figure 2). No periapical pathology was detected, and there was sufficient interradicular and apical bone (bone between the molar root tip and the inferior alveolar nerve). Hence, an immediate implant with SST and a customized healing abutment was planned. The patient was informed about the treatment plan and the prognosis, and an informed consent was obtained before starting the procedure. No written consent has been obtained from the patient as there is no patient identifiable data included in this case report.

### 2.1. Surgical Procedure

**Figure 3:**
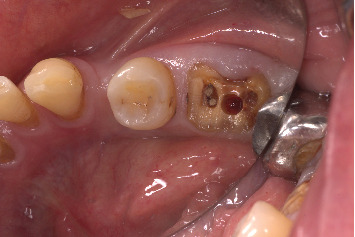
Root-guided osteotomy site for prosthetic positioning of implant.

**Figure 4:**
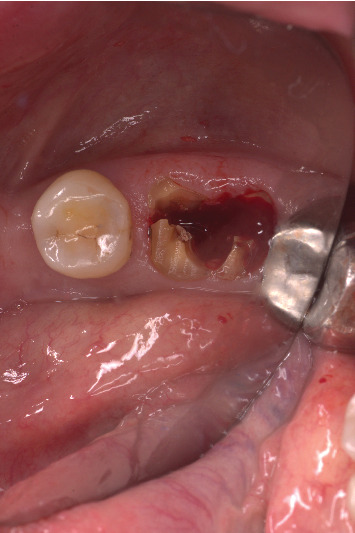
Separation of the buccal and lingual fragments of the roots.

**Figure 5:**
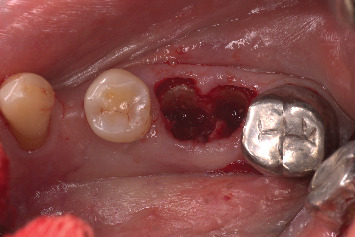
Atraumatic extraction of the lingual roots.

**Figure 6:**
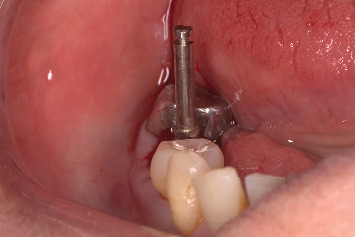
Ensuring parallelism prior to implant placement.

**Figure 7:**
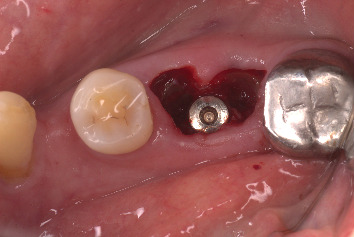
Implant placement.

**Figure 8:**
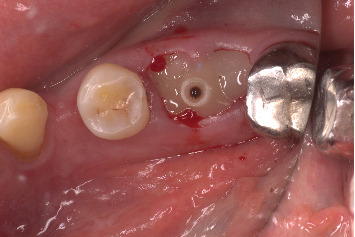
Fabrication of customized healing abutment with composite on polyoxymethylene abutment.

**Figure 9:**
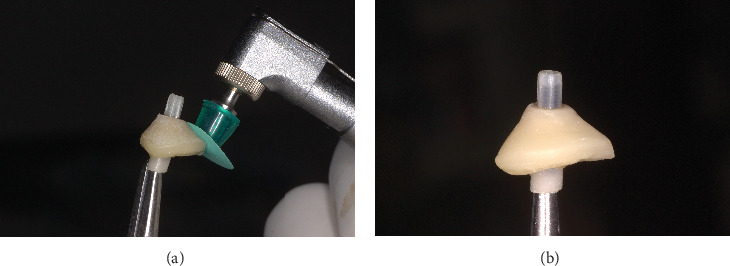
(a) Polishing the composite on the temporary abutment. (b) Customized healing abutment ready for insertion.

**Figure 10:**
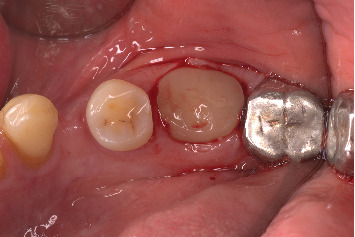
Customized healing abutment inserted.

The procedure was done flapless under local anesthesia. Root-guided osteotomy was done (Figure 3) for prosthetically guided implant placement. The root length was measured with an apex locator and Number 20 reamer. A gated glidden drill was marked for the measured length and used in a slow-speed hand piece to reach the apex and remove the gutta-percha completely. A long-shank carbide bur (Komet) was then used to separate the buccal and lingual sections of the roots mesiodistally (Figure 4). The lingual fragment of the root was atraumatically extracted (Figure 5). The buccal shield was adjusted with a round diamond bur to the level of the alveolar crest ensuring it was not mobile. An internal bevel was made on the coronal part of the shield for the prosthetic space. The socket was curreted thoroughly and cleaned with copious saline irrigation. Sequential osteotomy was done and parallelism confirmed (Figure 6). A 4.5 × 10 mm bone level implant (Bredent Medical, blueSKY) was placed with torque > 40 Ncm, not engaging the buccal shield (Figure 7). A customized healing abutment was fabricated using a polyoxymethylene temporary abutment (Bredent Medical, SKY Temp) and adding flowable composite to it gradually (Figure 8). The composite was highly polished using abrasive disk (Shofu Inc.) (Figures 9(a) and 9(b)) so that it does not accumulate plaque and facilitates positive gingival response. The customized healing abutment was inserted at a torque of 18 Ncm, and occlusal opening was sealed with Teflon and composite (Figure 10).  Appendix A illustrates the step-by-step procedure and armamentarium of SST typically on an anterior tooth. The same steps are applicable to a posterior tooth.

### 2.2. Prosthetic Phase

**Figure 11:**
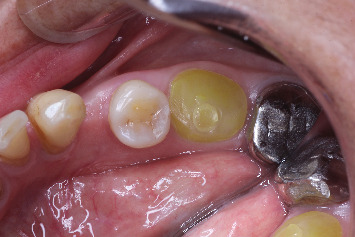
Recall at 4 months showing maintained buccal contour and positive gingival response.

**Figure 12:**
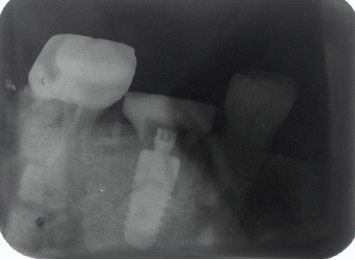
Intraoral periapical radiograph at the 4-month follow-up.

**Figure 13:**
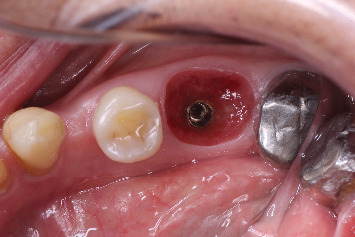
Healthy gingival cuff.

**Figure 14:**
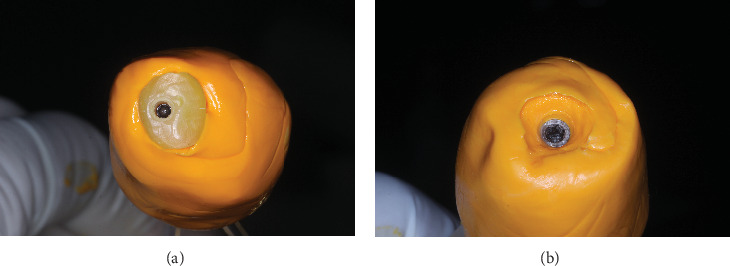
(a) Putty index for customizing impression coping. (b) Putty index for customizing impression coping.

**Figure 15:**
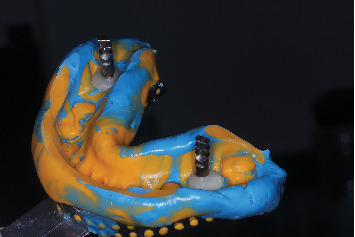
One-step light body-putty impression with definitive abutment used as customized impression coping.

**Figure 16:**
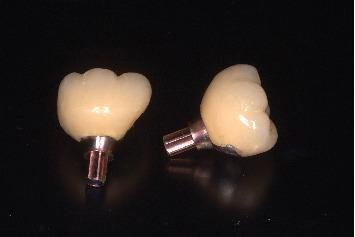
Screw-cement–retained PFM crown.

**Figure 17:**
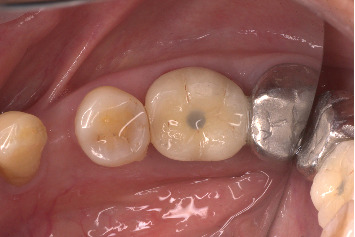
Abutment torqued and screw access hole sealed with composite.

**Figure 18:**
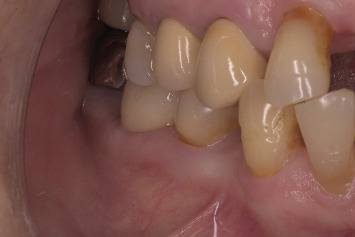
Occlusion checked.

The patient was recalled at 4 months for fabrication of the crown (Figure 11). Intraoral periapical radiograph revealed good bone formation around the implant (Figure 12). Removal of the customized healing abutment showed a healthy gingival cuff (Figure 13). The gingival profile was transferred (Figures 14(a) and 14(b)) with a multifunctional abutment (Bredent Medical, Exso Abutment) modified as customized impression coping (Figure 15), and a silicone final impression was made. A screw-retained porcelain-fused-to-metal (PFM) crown was fabricated (Figure 16) on the final abutment (Bredent Medical, SKY Abutment). The abutment was torqued to 25 Ncm, and the screw access hole was sealed with composite (Figure 17). The occlusion was checked (Figure 18) to ensure no eccentric contacts.

**Figure 19:**
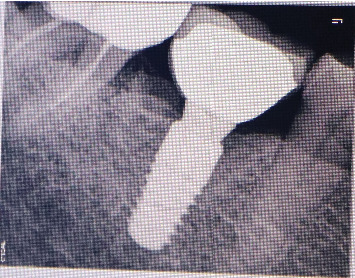
Two-year follow-up showing optimal bone levels.

**Figure 20:**
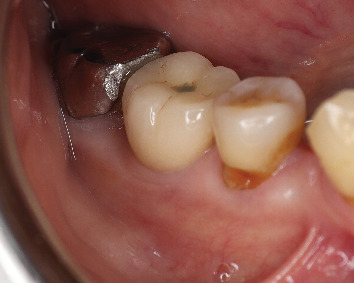
Two-year follow-up clinically showing buccal contour maintained.

A 2-year follow-up radiovisiograph (RVG) (Figure 19) shows maintained bone levels, and clinically, the soft tissue buccal contour and esthetics are maintained (Figure 20).

## 3. Discussion

Alveolar ridge resorption has long been considered an unavoidable consequence of tooth extraction and can be a significant problem in implant and restorative dentistry [10]. Socket shield aims to prevent such consequences by preserving the periodontal ligament–mediated blood supply to the labial cortical plate [4]. In the current case, SST was applied with an immediate implant to preserve the buccal periodontal structure, ensuring long-term success and reducing treatment time for the patient. A flapless approach was used, which further ensures better blood supply to the surgical area during the healing phase. When flap reflection is done, it entails a loss of the blood supply of the supraperiosteal vessels, so the bone vascularization depends upon its own vessels, which are a poor blood source in the case of cortical bone. This will imply a certain level of bone resorption during healing in cases that occur with a mucoperiosteal flap reflection [11]. Hence, an SST with immediate implant done by a flapless approach minimizes the surgical trauma, reduces treatment time, improves esthetics, and thus ultimately leads to increased patient satisfaction.

There is no general agreement on whether grafting with biomaterials should be mandatory after immediate implant placement using the SST. In the present case, no grafting was performed at the mandibular molar site. A histological study of a human specimen suggested that while grafting material might be beneficial, it could also increase the risk of infection and delay healing. The study found a significant amount of mature, dense bone in the apical and middle regions of the gap between the membrane and the implant surface, and noted that achieving bone of this quality with grafting material would be challenging. Additionally, the connective tissue in the specimen was noninfiltrated, and the lack of inflammation in this area was viewed as a positive outcome [12]. A study evaluated the radiographic and clinical findings in SST without graft and concluded that the SST without the use of bone grafts appears to yield successful clinical and esthetic outcomes. Avoiding the use of bone grafts could increase the cost-effectiveness of this treatment approach [13]. Other researchers have also produced successful results without the use of any grafting materials and believe that new bone is formed in the space rather than in cementum [14, 15]. However, other researchers prefer to fill the jumping gap with xenograft to prevent any soft tissue migration within the space [16, 17].

A customized healing abutment was used to enhance the gingival profile and maintain the interdental papilla levels. It also served the purpose of protecting the implant site during the healing phase as it was a flapless implant placement and primary closure was difficult. Customized healing abutments have been introduced in clinical practice along with implant surgery to preserve or create natural-appearing hard and soft tissues around the implant. This provides the benefits of reducing the overall treatment time by eliminating the second-stage surgery for reopening the implant site. This, in turn, reduces the time for the fabrication of the final prostheses. The customized healing abutment protects the implant site during the initial postsurgical healing stage from the accumulation of plaque or debris [18].

Pohl et al. [8] investigated sockets treated with a socket shield, allowing them to heal either with a blood clot or grafted using autologous materials, including autologous platelet-rich fibrin (PRF), scraped particulate bone, cortical tuberosity bone plate, or particulate dentin, all covered with PRF membranes. Four months after the initial surgery, the sites were exposed via flap, and the depth and width of the soft tissue ingrowth next to the root fragment were measured using a scaled probe. The study of 34 sites found that the greatest soft tissue ingrowth occurred in the nongrafted sockets (6.0 ± 0.0 mm). Grafting with PRF plugs (2.3 ± 0.2 mm) or particulate bone (2.7 ± 0.6 mm) resulted in reduced soft tissue ingrowth. The smallest depth of the soft tissue ingrowth, at 1 mm, was observed in sockets grafted with particulate dentin or cortical tuberosity bone plate, which yielded the best clinical outcome. It is important to note that the nongrafted sites were left uncovered.

In this case, the socket opening was sealed with a customized healing abutment, which may act as a contact inhibitor of epithelial cell growth (restorative tissue inhibitor (RTI)), likely due to blood clot stability and hence, reduce the chances of soft tissue ingrowth. This hypothesis, however, needs scientific proof, including histological data [19].

Managing postextraction ridge changes in the posterior region by prevention or regeneration remains a challenge. The socket shield aims to offset these ridge changes wherever possible, preserving the patient's residual tissues at immediate implant sites [20]. A recent meta-analysis found that, although based on limited evidence, SST was more effective than conventional immediate implant placement in reducing bone resorption, enhancing implant stability, and improving esthetic results. However, due to the absence of a standardized surgical approach, SST cannot be recommended as a routine clinical practice at this time. More high-quality randomized controlled trials (RCTs) are needed to confirm these findings [21].

### 3.1. Clinical Recommendations

1. Case selection is very important for successful execution of the technique and to ascertain long-term results. Avoid cases with any unresolved pathology present.
2. Clinically check for any mobility of the shield. If the buccal shield is mobile during the procedure, it is recommended to abort SST, extract the mobile root fragment, and proceed with immediate implant placement with or without GBR as per clinical condition.
3. SST is a minimally invasive option not only for the anterior zone but also for the posterior region.
4. The customized healing abutment should be highly polished to avoid any plaque accumulation.
5. SST is a technique-sensitive procedure, requiring specific armamentarium plus a good surgical and prosthetic skill set.

## 4. Conclusion

SST is a promising, noninvasive technique for preserving hard and soft tissues during immediate implant placements. A customized healing abutment further enhances the soft tissue emergence profile. This combined approach reduces the overall treatment time and provides a viable cost-effective option to the patient for replacing nonrestorable teeth. Long-term RCTs focusing on posterior sites need to be conducted to provide supportive data for this technique before it can become a routine procedure in clinical practice.

## Figures and Tables

**Figure 21:**
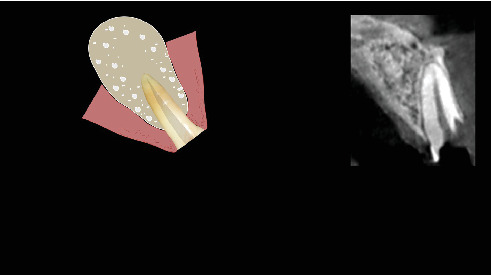
Fractured nonrestorable tooth.

**Figure 22:**
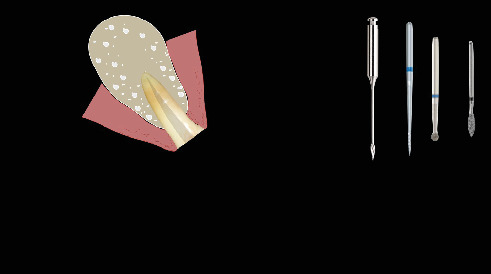
Armamentarium required.

**Figure 23:**
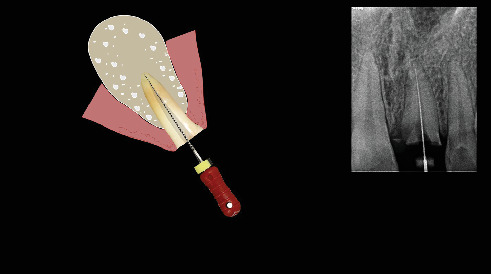
Determine length of the root.

**Figure 24:**
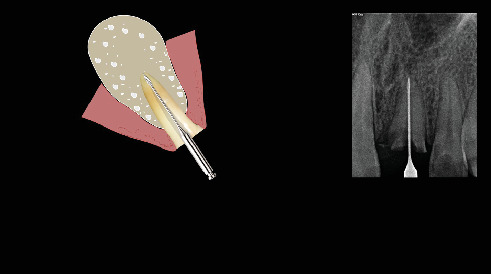
Reach the apex with gates glidden drill and confirm that on radiograph.

**Figure 25:**
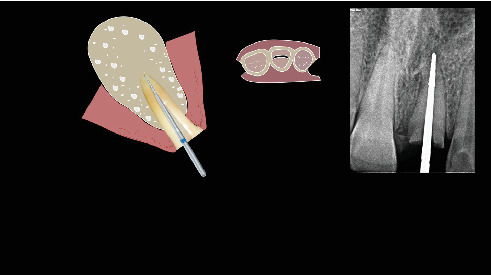
Split the root mesiodistally till apex.

**Figure 26:**
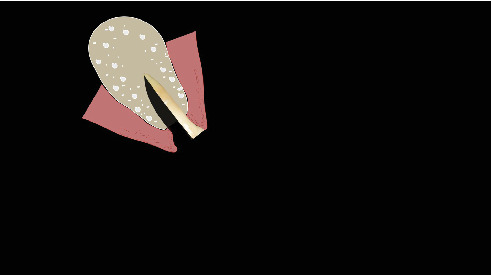
Removal of palatal segment.

**Figure 27:**
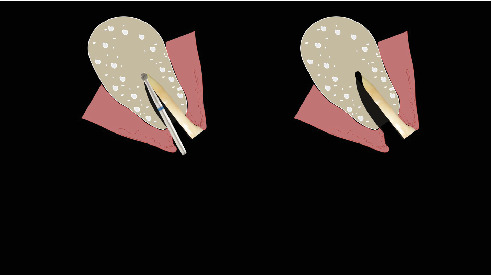
Trim the apex with a round bur.

**Figure 28:**
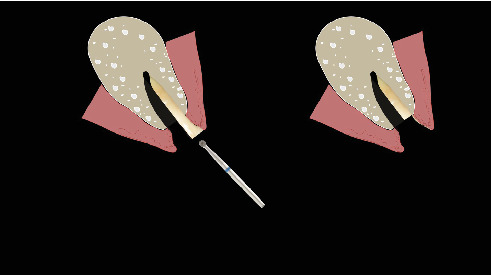
Trim the shield till alveolar crest.

**Figure 29:**
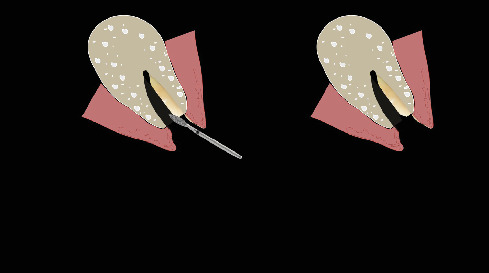
Create an internal bevel for prosthetic space.

**Figure 30:**
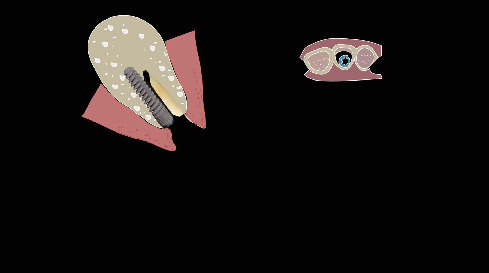
Immediate implant placement engaging the apical and palatal bone.

**Figure 31:**
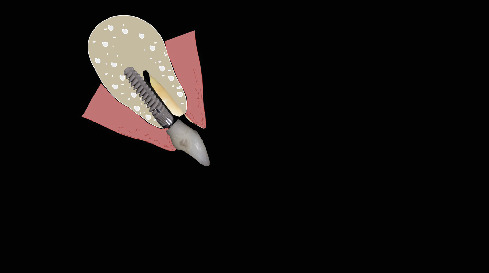
Immediate implant placement and provisionalization.

## Data Availability

The figures and appendices data used to support the findings of this study are included within the article.
